# Baicalin Mitigates the Neuroinflammation through the TLR4/MyD88/NF-*κ*B and MAPK Pathways in LPS-Stimulated BV-2 Microglia

**DOI:** 10.1155/2022/3263446

**Published:** 2022-11-09

**Authors:** Baojing Li, Mingming Wang, Shuai Chen, Manping Li, Jing Zeng, Saichun Wu, Yuanqing Tu, Yanping Li, Rongping Zhang, Feng Huang, Xiaoyun Tong

**Affiliations:** ^1^The First Affiliated Hospital of Yunnan University of Chinese Medicine, Yunnan University of Chinese Medicine, Kunming, China; ^2^Yunnan Key Laboratory of Southern Medicinal Utilization, College of Traditional Chinese Medicine, Yunnan University of Chinese Medicine, Kunming, China; ^3^State Key Laboratory of Quality Research in Chinese Medicine, Macau University of Science and Technology, Macau, China; ^4^College of Pharmacy, Jinan University, Guangzhou, China

## Abstract

Baicalin (BA) is a major flavone from *Scutellaria baicalensis* Georgi and has showed significant curative effects in Parkinson's and Alzheimer's diseases. In the present study, we investigated the effects of BA on antineuroinflammation and related signaling cascade in lipopolysaccharide- (LPS-) induced BV-2 microglial model. The results showed that BA significantly attenuated inflammatory mediators (NO, iNOS, IL-1*β*, COX-2, and PGE2) and suppressed the expression of miR-155. More crucially, BA could regulate the expression of related proteins in Toll-like receptor 4 (TLR4)/myeloid differentiation protein 88 (MyD88)/nuclear factor *κ*B (NF-*κ*B) pathway and suppress the phosphorylation of mitogen-activated protein kinase (MAPK) family. In addition, molecular docking analysis indicated that BA binds to the amino acids Lie 63 and Tyr 65 of TLR4 by *π*-*σ* and *π*-*π* T-shaped interaction. Thus, BA suppressed the LPS-stimulated neuroinflammation in BV-2 microglia by blocking the TLR4-mediated signal transduction through TLR4/MyD88/NF-*κ*B and MAPK pathways and inhibiting the miR-155 expression. Our findings demonstrated that BA could be a valuable therapeutic for the treatment of neuroinflammation and neurodegenerative diseases.

## 1. Introduction

Neurodegenerative disease characterized by progressive neuronal death is a kind of chronic disease of the central nervous system (CNS) including Alzheimer's disease (AD), Parkinson's disease (PD), and Huntington disease (HD) [1]. Studies have shown that chronic inflammation in the brain may be one of the essential pathological features of neurodegenerative diseases [2]. Microglial cells are resident immunocompetent cells of CNS and play a crucial role in the brain injury and development of neurodegenerative diseases. Under normal physiological conditions, resting microglia are pivotal players in nourishing, supporting, and protecting neurons [3]. However, when there is a neuronal injury or other stimuli, such as lipopolysaccharide (LPS), microglia will be activated to secrete massive proinflammatory and cytotoxic factors, including nitric oxide (NO), prostaglandin E2 (PGE2), interleukin 1*β* (IL-1*β*), and peroxides, which ultimately leads to the cell death [4]. Excessive activation of microglia can damage the surrounding normal neural tissue, and the inflammatory factors secreted by the dying neurons in turn aggravate the chronic activation of microglia resulting in the gradual loss of neurons. Such case was observed in AD, PD, and HD [5, 6]. Studies have shown that overactivated microglia also highly express Toll-like receptor 4 (TLR4) and miR-155 [7]. LPS binds and activates TLR4, leading to the sequential recruitment/activation of the myeloid differentiation protein 88 (MyD88) and interleukin-1 receptor-associated kinases (IRAKs). Stimulation of TLR4 also activates mitogen-activated protein kinase (MAPK) and the nuclear factor-*κ*B (NF-*κ*B) pathways, which then evokes the inflammatory response in microglia [8]. Thus, control of LPS-induced microglia activation, suppression of the production of neurotoxic proinflammatory mediators and cytokines, and downregulation of proteins involved in TLR4-mediated signaling pathways would be effective therapeutic strategies for neuroinflammatory diseases.

**Figure 1:**
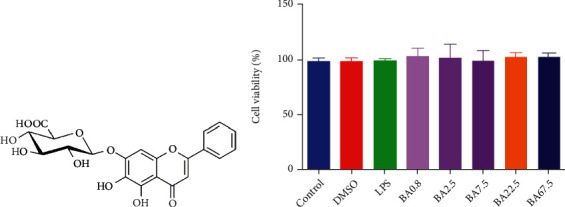
Cytotoxicity of BA in BV-2 microglia. The chemical structure of BA (a). The effect of BA on the viability of BV-2 cells (b). BV-2 cells were incubated for 24 h in the presence of the indicated concentrations of BA (0.8-67.5 *μ*M). Results were presented as mean ± SD (*n* = 6).

Baicalin (baicalein 7-*O*-*β*-D-glucopyranosiduronate, BA, chemical structure shown in Figure 1(a)) is a major active flavone isolated from the Radix of *Scutellaria baicalensis* Georgi. It has obvious curative effect in CNS diseases. Studies have shown that BA displays neuroprotective effect on permanent cerebral ischemia injury in rats through downregulating the expression of inducible nitric oxide synthase (iNOS) mRNA, COX-2 mRNA, and cleaved caspase-3 protein [9]. BA can also reduce amyloid *β* protein-induced neurotoxicity in PC12 cells [10]. In addition, BA exerts anti-inflammatory effect by inhibiting the NF-*κ*B signaling cascade in *Helicobacter pylori*-induced gastritis model [11]. These researches indicate that BA plays a key role in the treatment of neuroinflammatory and neurodegenerative diseases. However, the pharmacological effects of BA on activated microglia have not been elucidated. Thus, our study evaluated the neuroinflammatory-modulatory effects and the underlying mechanisms of BA in LPS-stimulated microglia.

## 2. Materials and Methods

### 2.1. Chemicals and Reagents

Baicalin was obtained from Nanjing Zelang Medical Technology. LPS, dimethyl sulfoxide (DMSO), 3-(4, 5-dimethylthiazol-2-yl)-2,5-diphenyltetrazolium bromide (MTT), indomethacin (INDO), dexamethasone (DEX), aminoguanidine (AG, iNOS inhibitor), NS-398 (COX-2 inhibitor), and pyrrolidine dithiocarbamate (PDTC, I*κ*B*α*, and NF-*κ*B P65 inhibitor) were purchased from Sigma-Aldrich. NO and iNOS assay kits were purchased from Nanjing Jiancheng Bioengineering Institute. Real-time PCR kit was purchased from Shanghai Gene Pharma. PGE2 and IL-1*β* ELISA kits were purchased from R&D Systems. MAPK family antibody sampler kit, phosphorylated MAPK family antibody sampler kit, and primary antibodies against *β*-actin, COX-2, IRAK1, IRAK4, MyD88, and NF-*κ*B P65 were purchased from Cell Signaling Technology. Interleukin-1 receptor-associated kinase 1/4 inhibitor (IRAK1/4 inhibitor), TLR4, P-I*κ*B*α*, and I*κ*B*α* antibodies were purchased from Santa Cruz Biotechnology. MTS510 (TLR4 inhibitor) was purchased from eBioscience. SB203580 (P38 MAPK inhibitor), SP600125 (JNK inhibitor), ST2825 (MyD88 inhibitor), and U0126 (ERK inhibitor) were purchased from the Beyotime Institute of Biotechnology. RPMI 1640 and fetal bovine serum (FBS) were purchased from Gibco.

### 2.2. Cell Culture

BV-2 was purchased from Cell Centre of Peking Union Medical College (Beijing, China). The cells were cultured in RPMI 1640 including 10% FBS, 100 U/mL penicillin, and 100 *μ*g/mL streptomycin (conditions: 5% CO_2_ at 37°C).

### 2.3. Cell Viability Assay

Cell viability was determined by the MTT assay. Briefly, BV-2 (5 × 10^4^/well) was inoculated into 96-well plates and cultured for 24 h. Then, various concentrations of BA (0.8-67.5 *μ*M) were added into the wells for 24 h. Following the incubation, the supernatant was substituted for the same volume of fresh serum-free RPMI-1640 for 24 h. The cells were then cultured with 0.5 mg/mL MTT for another 4 h. Subsequently, 150 *μ*L DMSO was added and incubated for 10 min. The absorbance was measured with a spectrophotometer at 570 nm.

### 2.4. NO and iNOS Assays

The content of NO and iNOS secreted by microglia was measured using NO assay kit and iNOS assay kit, respectively. Briefly, BV-2 (5 × 10^5^ cells/mL) was cultured in 24-well plates overnight and pretreated with BA at the concentration of 2.5, 7.5, and 22.5 *μ*M for 1 h. The cells were then incubated with LPS (0.1 *μ*g/mL) for 12 h. Indomethacin (INDO, 10 *μ*M) and aminoguanidine (AG, 1 mM) were added as positive control. The supernatant was collected; the absorbance of NO and iNOS was measured at 550 nm and 530 nm.

### 2.5. Measurement of IL-1*β* and PGE2 Production

The levels of the PGE2 and IL-1*β* in culture medium were assessed using ELISA assay kits. BV-2 (5 × 10^5^ cells/mL) was cultured in 24-well plates for overnight and pretreated with BA at the concentration of 2.5, 7.5, and 22.5 *μ*M for 1 h. The cells were incubated with LPS (0.1 *μ*g/mL) for 24 h. Dexamethasone (DEX, 10 *μ*M) and indomethacin (INDO, 10 *μ*M) were added as positive control. Absorbance was measured at 450 nm using a spectrometer.

### 2.6. Real-Time PCR Analysis

Real-time PCR was carried out to analyze the mRNA level of miR-155. BV-2 (5 × 10^5^ cells/mL) was cultured in 6-well plates overnight and pretreated with BA (2.5, 7.5, and 22.5 *μ*M) for 1 h. The cells were incubated with LPS (0.1 *μ*g/mL) for 12 h. The supernatant was removed, and total RNA was extracted by TRIzol reagent following the manufacturer's instructions. The total RNA was quantitatively analyzed and then primed with mmu-miR-155-5p-real-time-F: 5′-CGGCGGTTAATGCTAATTGTGAT-3′ and mmu-miR-155-5p-real-time-R: 5′-GTGCAGGGTCCGAGGT-3′ to synthesize cDNA using M-MLV reverse transcriptase. PCR was performed by 40 cycles of denaturation at 95°C for 3 min, annealing at 62°C for 50 s, and extension at 95°C for 50 s. Indomethacin (INDO, 10 *μ*M) and dexamethasone (DEX, 10 *μ*M) were added as positive control.

### 2.7. Western Blot Analysis

BV-2 (5 × 10^5^ cells/mL) was cultured in 6-well plates for 12 h, followed by the treatment with BA (2.5, 7.5, and 22.5 *μ*M) for 1 h. The cells were incubated with LPS (0.1 *μ*g/mL) for appropriate time; iNOS, COX-2, TLR4, and MyD88 were stimulated for 12 h, IRAK1 and IRAK4 for 20 min, p-ERK/ERK for 10 min, p-JNK/JNK and p-P38 MAPK/P38 MAPK for 40 min, p-I*κ*B*α*/I*κ*B*α*for 15 min, and NF-*κ*B P65 for 1 h. The following cells were washed with PBS three times and lysed in 100 *μ*L of RIPA lysis buffer on ice for 30 min. Lysates were then centrifuged at 10,000 × *g* at 4°C for 10 min. Approximately 20 *μ*L of the supernatant containing 50 *μ*g total protein was subjected to 8–10% sodium dodecyl sulfate-polyacrylamide gel electrophoresis (SDS-PAGE) and subsequently transferred to polyvinylidene fluoride (PVDF) membranes and incubated with the appropriate primary antibodies against iNOS, COX-2, TLR4, MyD88, IRAK1, IRAK4, ERK, p-ERK, JNK, p-JNK, P38 MAPK, p-P38 MAPK, I*κ*B*α*, p-I*κ*B*α*, and NF-*κ*B P65. Membranes were then incubated with secondary antibodies combined to horseradish peroxidase for appropriate time. After washing three times in Tris-buffered saline with Tween 20 (TBST), the protein bands were visualized using the enhanced chemiluminescence reagents.

### 2.8. Molecular Docking Analysis

The crystal structure of TLR4-myeloid differential protein-2 (MD2) complex (PDB ID 3FXI, chain A (605 residues), and C (142 residues)) was obtained from the Protein Data Bank (PDB) (http://www.wwpdb.org/). The structure of BA was sketched in ChemDraw 20.0 and saved as Mol2 format. The TLR4-MD2 crystal structure was optimized by Pymol software following the removal of water molecules and ligands, the addition of missing residues [12]. Molecular docking scores and poses were calculated in Autodock Tools 1.5.6 [13]. All rotatable bonds in ligand remained flexible, while the protein structure kept rigid. In the docking setup, binding site center was set up to *x* = 20.000, *y* = −14.111, and *z* = 12.000; the spacing box was 0.369 Å; and the number of points in dimension was set up to *x* = 88, *y* = 86, and *z* = 92. Molecular interactions of TLR4-MD2 ligand complexes were identified with Discovery Studio 2016 software [14]. The interaction energy of the TLR4-MD2 ligand complex was calculated, 3D and 2D diagrams of interaction were created, and their interactions include van der Waals force, hydrogen bond, and *π*-*σ* interaction.

### 2.9. Statistical Analysis

Results were analyzed by GraphPad Prism 6.0 software (GraphPad, United States). The data were expressed as means ± SD of three independent experiments performed in triplicate measurements. Statistical analysis was performed using ANOVA followed by Tukey's test. *P* < 0.05 was considered as statistical significance.

## 3. Results

### 3.1. BA Does Not Affect Cell Viability in Microglia

MTT assay showed that BA had no obvious effect on the viability of BV-2 cells in concentration range from 0.8 *μ*M to 67.5 *μ*M (Figure 1(b)). Therefore, we used BA at concentration range from 2.5 to 22.5 *μ*M for subsequent studies.

### 3.2. BA Inhibits the Production of NO and iNOS in LPS-Induced Microglia

**Figure 2:**
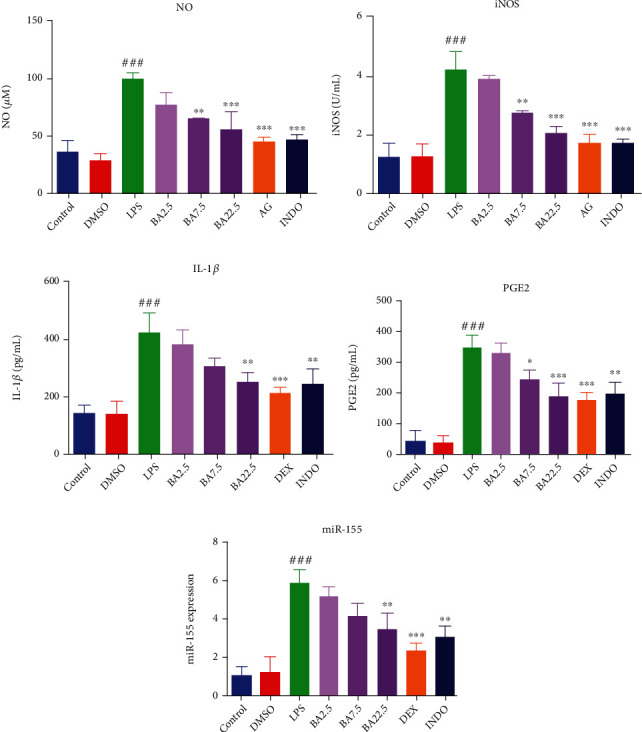
BA inhibits the production and expression of inflammatory molecules in LPS-stimulated BV-2 microglia. The effects of BA on NO production (a), iNOS activity (b), IL-1*β* (c), and PGE2 (d) production in LPS-stimulated BV-2 cells. BV-2 cells were incubated for 1 h in the presence of the indicated concentrations of BA (2.5-22.5 *μ*M). Then, the cells were stimulated with or without LPS (0.1 *μ*g/mL); the production of NO and iNOS (incubated for 12 h) and IL-1*β* and PGE2 (incubated for 24 h) was determined by ELISA kit. MiR-155 expression (e) was detected by miRNA real-time PCR kit, BV-2 was pretreated with BA (2.5-22.5 *μ*M) for 1 h followed by LPS stimulation for 12 h, and then, RT-PCR was performed. Results were presented as mean ± SD (*n* = 3). ^###^*P* < 0.001 vs. control and ^∗^*P* < 0.05, ^∗∗^*P* < 0.01, and ^∗∗∗^*P* < 0.001 vs. LPS-treated cells.

NO and iNOS are the major inflammatory mediators in microglia. Activation of microglial cells was closely associated with the expression of iNOS, while iNOS mediates the synthesis of NO. As expected, LPS significantly increased the production of NO and iNOS in microglia (Figures 2(a) and 2(b)). NO level in LPS-induced microglia treated with 2.5, 7.5, and 22.5 *μ*M of BA was reduced by 22.43%, 34.79%, and 44.14%, respectively (Figure 2(a)). The iNOS level in LPS-induced microglial cells treated with 2.5, 7.5, and 22.5 *μ*M of BA was reduced by 7.21%, 34.77%, and 50.92%, respectively (Figure 2(b)). These results indicated that BA inhibits the expression of NO and iNOS in LPS-stimulated microglia in a dose-dependent manner.

### 3.3. BA Inhibits the Production of Proinflammatory Cytokines IL-1*β* and PGE2 in LPS-Induced Microglia

IL-1*β* and PGE2 are important proinflammatory cytokines. Production of IL-1*β* in the microglia is the initiating step of nerve inflammation, which drives the amplification of inflammatory cascade and pathogenesis of neurodegenerative disease. PGE2 is the metabolite of arachidonic acid generated by rate-limiting enzyme cyclooxygenase-2 (COX-2), and it is another important indicator of varied inflammatory diseases [15]. The inhibitory effects of BA on proinflammatory cytokines IL-1*β* and PGE2 were determined using ELISA. As expected, treatment of BV-2 with LPS resulted in a significant increase of IL-1*β* and PGE2 release compared with control. IL-1*β* level in LPS-induced microglial cells treated with 2.5, 7.5, and 22.5 *μ*M of BA was reduced by 9.71%, 27.79%, and 40.53%, respectively (Figure 2(c)). PGE2 level in LPS-induced microglial cells treated with 2.5, 7.5, and 22.5 *μ*M of BA was reduced by 3.11%, 28.45%, and 44.95%, respectively (Figure 2(d)). These results indicated that BA inhibits the release of IL-1*β* and PGE2 in LPS-stimulated microglia in a dose-dependent manner.

### 3.4. BA Downregulates the Expression of miR-155 in LPS-Induced Microglia

Studies have shown that LPS can stimulate the overexpression of TLR4 in microglia, and overexpression of TLR4 is positively correlated with the upregulation of miR-155 through MyD88 signaling pathway [16, 17]. We evaluated the effects of BA on the expression of miR-155 by real-time PCR. As expected, treatment of BV-2 cells with LPS resulted in a significant increase of miR-155 level compared with control. The level of miR-155 in LPS-induced microglia treated with 2.5, 7.5, and 22.5 *μ*M of BA was reduced by 11.84%, 28.90%, and 33.55%, respectively (Figure 2(e)). These results indicated that BA downregulates the expression of miR-155 in LPS-stimulated microglia in a dose-dependent manner.

### 3.5. BA Inhibits the Protein Expression of iNOS and COX-2 in LPS-Stimulated Microglia

**Figure 3:**
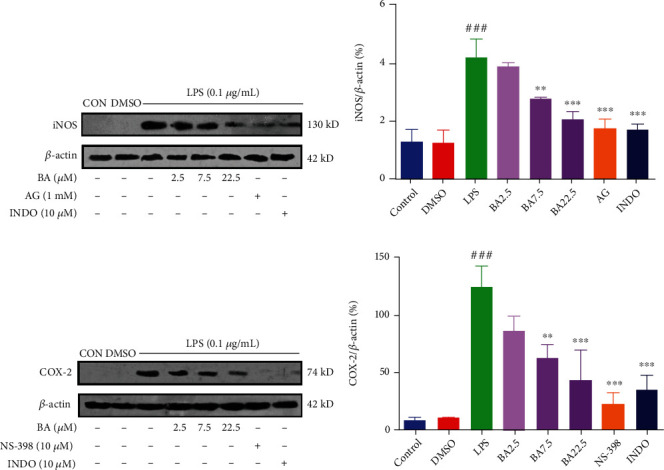
BA inhibits the expression of iNOS and COX-2 in LPS-stimulated BV-2 microglia. The effects of BA on the expression of iNOS (a, b) and COX-2 (c, d) in LPS-stimulated in BV-2 cells. BV-2 cells were incubated for 1 h in the presence of the indicated concentrations of BA (2.5-22.5 *μ*M). Then, the cells were stimulated with or without LPS (0.1 *μ*g/mL) for 12 h. After stimulation, proteins were extracted and analyzed by Western blot. Results were obtained from three independent experiments performed in triplicate, and values are mean ± SD (*n* = 3). ^###^*P* < 0.001 vs. control and ^∗∗^*P* < 0.01 and ^∗∗∗^*P* < 0.001 vs. LPS-treated cells.

To further determine if the inhibition of NO and PGE2 expression by BA is related with the decrease of iNOS and COX-2 protein levels, we performed Western blot experiments. As expected, iNOS and COX-2 protein levels were increased after LPS stimulation. The protein level of iNOS in LPS-induced microglial cells treated with 2.5, 7.5, and 22.5 *μ*M of BA was reduced by 16.02%, 27.33%, and 52.47%, respectively. As positive controls, iNOS inhibitor AG (1 mM) and INDO (10 *μ*M) also reduced the level of iNOS by 59.69% and 52.37%, respectively (Figures 3(a) and 3(b) and Figure S1 in Supplementary Materials). The protein level of COX-2 in LPS-induced microglial cells treated with 2.5, 7.5, and 22.5 *μ*M of BA was reduced by 30.49%, 48.99%, and 64.35%, respectively. As positive controls, COX-2 inhibitor NS-398 (10 *μ*M) and INDO (10 *μ*M) also reduced the level of COX-2 by 81.20% and 71.39%, respectively (Figures 3(c) and 3(d) and Figure S2 in Supplementary Materials). These results indicate that BA inhibits the expression of COX-2 and iNOS in LPS-stimulated microglia in a dose-dependent manner.

### 3.6. BA Inhibits TLR4/NF-*κ*B Pathway in LPS-Stimulated Microglia

**Figure 4:**
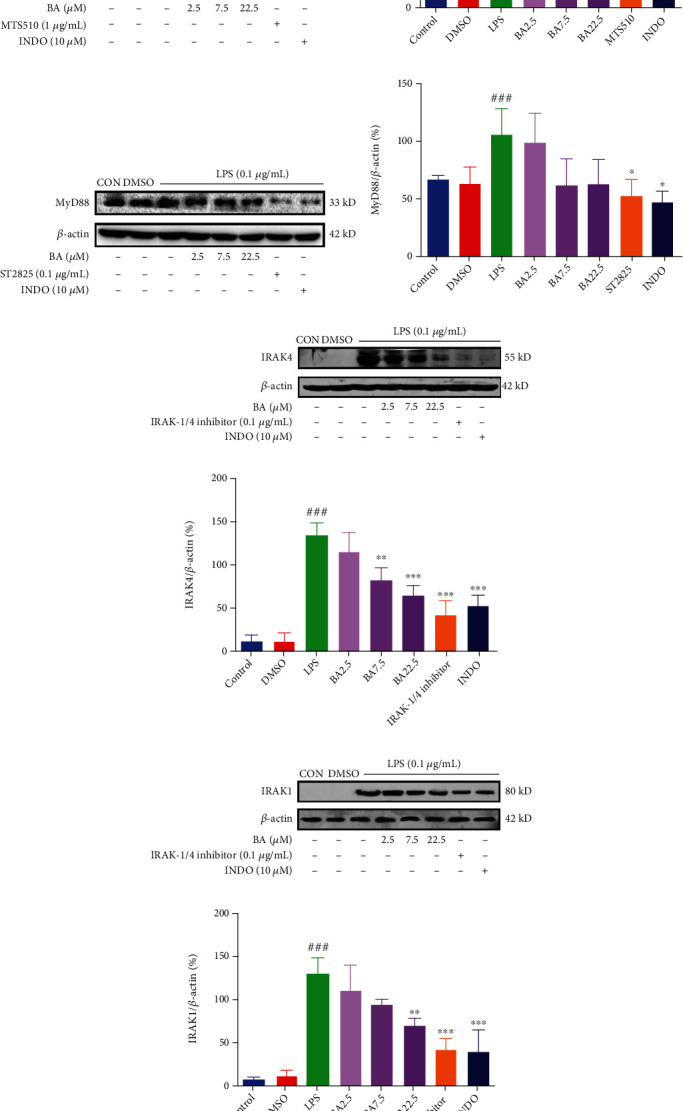
Effects of BA on TLR4 pathways in LPS-stimulated BV-2 microglia. The expression levels of TLR4 (a, b), MyD88 (c, d), IRAK4 (e, f), and IRAK1 (g, h) were analyzed by Western blot. BV-2 was pretreated with BA (2.5-22.5 *μ*M) for 1 h before being stimulated with 0.1 *μ*g/mL LPS for different times (12 h for TLR4 and MyD88 and 20 min for IRAK4 and IRAK1). Results were presented as mean ± SD (*n* = 3). ^###^*P* < 0.001 vs. control; ^∗^*P* < 0.05, ^∗∗^*P* < 0.01, and ^∗∗∗^*P* < 0.001 vs. LPS.

**Figure 5:**
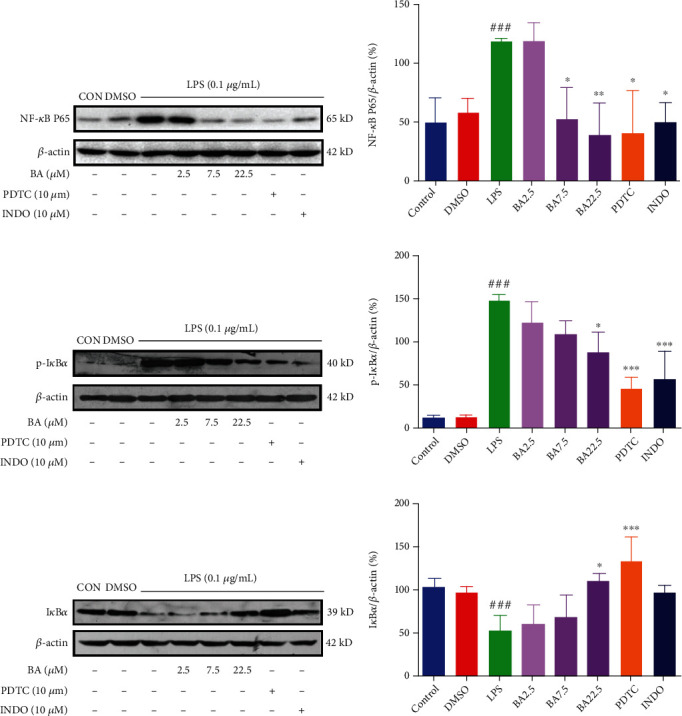
Effects of BA on NF-*κ*B pathway in LPS-stimulated BV-2 microglia. The expression levels of I*κ*B*α* (a, b), p-I*κ*B*α* (c, d), and NF-*κ*B P65 (e, f) were analyzed by Western blot. BV-2 was incubated with BA (2.5-22.5 *μ*M) for 1 h before being stimulated with 0.1 *μ*g/mL LPS for different times (15 min for I*κ*B*α* and P-I*κ*B*α* and 1 h for NF-*κ*B p65). Results were presented as mean ± SD (*n* = 3). ^#^*P* < 0.05 and ^###^*P* < 0.001 vs. control; ^∗^*P* < 0.05, ^∗∗^*P* < 0.01, and ^∗∗∗^*P* < 0.001 vs. LPS.

To further clarify the role of BA, we carried out Western blot to determine the expression of proteins in TLR4/NF-*κ*B signaling pathway including TLR4, MyD88, IRAK4, IRAK1, I*κ*B*α*, P-I*κ*B*α*, and NF-*κ*B P65. As shown in Figures 4(a)–4(h), treatment with BA (at the concentration of 2.5, 7.5, and 22.5 *μ*M) downregulated the expression of TLR4 (by 19.75%, 25.11%, and 41.92%, respectively), MyD88 (by 7.43%, 42.59%, and 41.18%, respectively), IRAK4 (by 14.60%, 41.46%, and 51.93%, respectively), and IRAK1 (by17.40%, 28.13%, and 48.50%, respectively) in LPS-stimulated microglia cells (Figures S3–S6 in Supplementary Materials). As positive controls, TLR4 inhibitors MTS510 (1 *μ*g/mL) and INDO (10 *μ*M) downregulated the expression of TLR4 by 58.89% and 57.42%, respectively. MyD88 inhibitor ST2825 (0.1 *μ*g/mL) and INDO (10 *μ*M) decreased the expression of MyD88 by 51.68% and 55.96%, respectively. IRAK1/4 inhibitor (0.1 *μ*g/mL) and INDO (10 *μ*M) reduced the expression of IRAK4 by 68.73% and 61.35%, respectively. IRAK1/4 inhibitor (0.1 *μ*g/mL) and INDO (10 *μ*M) reduced the expression of IRAK1 by 66.23% and 69.04%, respectively. As shown in Figures 5(a)–5(f), treatment with BA (at the concentration of 2.5, 7.5, and 22.5 *μ*M) downregulated the expression of NF-*κ*B P65 (by 0.03%, 55.58%, and 67.57%, respectively) and p-I*κ*B*α* (by 16.95%, 26.54%, and 41.02%, respectively) in LPS-stimulated microglial cells (Figures S7–S8 in Supplementary Materials). In contrast, treatment with BA (at the concentration of 2.5, 7.5, and 22.5 *μ*M) increased the expression of I*κ*B*α* level (by 16.02%, 31.86%, and 109.81%, respectively) in LPS-stimulated microglial cells (Figure S9 in Supplementary Materials). As positive controls, the inhibitor PDTC (10 *μ*M) downregulated the expression NF-*κ*B P65 and p-I*κ*B*α* by 65.94% and 69.08%, respectively, and upregulated the expression of I*κ*B*α* by 155.05%. INDO (10 *μ*M) inhibited the expression of NF-*κ*B P65 and p-I*κ*B*α* by 61.28% and 62.00%, respectively, while increased the expression of I*κ*B*α* by 84.89%.

### 3.7. BA Inhibits the Phosphorylation of Proteins in MAPK Signaling in LPS-Stimulated Microglia

**Figure 6:**
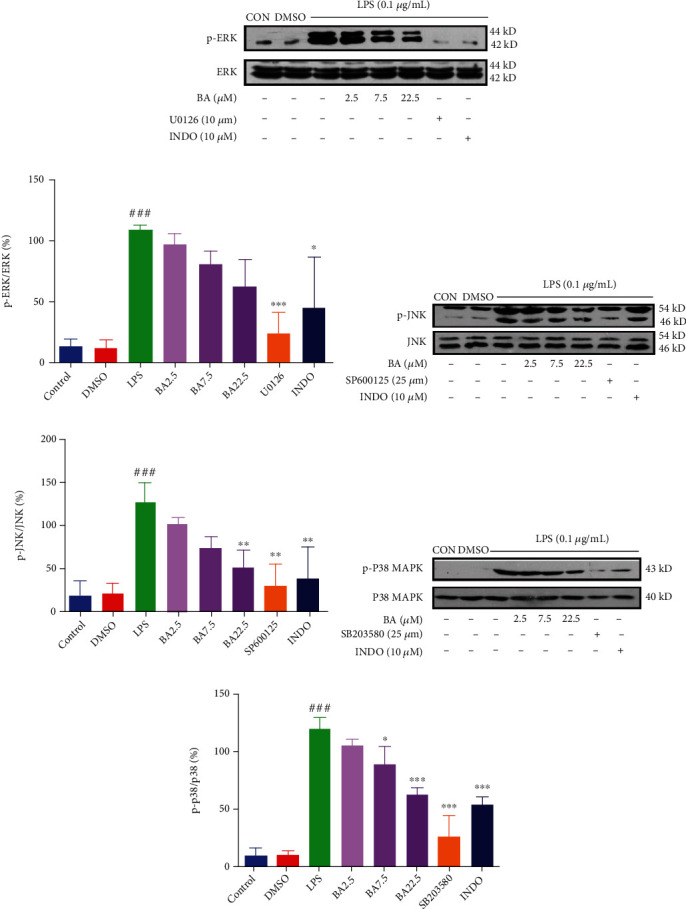
Effects of BA on MAPK pathways in LPS-stimulated BV-2 microglia. The expression levels of p-ERK and ERK (a, b), p-JNK and JNK (c, d), and p-P38 MAPK and P38 MAPK (e, f) were analyzed by Western blot. BV-2 was incubated with BA (2.5-22.5 *μ*M) for 1 h before being stimulated with 0.1 *μ*g/mL LPS for different times (10 min for p-ERK/ERK and 40 min for p-JNK/JNK and p-P38 MAPK/P38 MAPK). Results were presented as mean ± SD (*n* = 3). ^###^*P* < 0.001 vs. control; ^∗^*P* < 0.05, ^∗∗^*P* < 0.01, and ^∗∗∗^*P* < 0.001 vs. LPS.

To further assess the effect of BA, we analyzed the protein phosphorylation in MAPK signaling pathway. Treatment with BA (at the concentration of 2.5, 7.5, and 22.5 *μ*M) could suppress the level of p-ERK (by 10.66%, 26.41%, and 42.28%, respectively) (Figures 6(a) and 6(b)), p-JNK (by 20.11%, 41.44%, and 60.13%, respectively) (Figures 6(c) and 6(d)), and p-P38 MAPK (by 12.12%, 25.55%, and 47.52%, respectively) (Figures 6(e) and 6(f)) in LPS-stimulated microglia (Figures S10–S12 in Supplementary Materials). As positive controls using their respective inhibitors, U0126 (10 *μ*M) decreased the level of p-ERK by 76.93%, SP600125 (25 *μ*M) downregulated the level of p-JNK by 76.18%, and SB203580 (25 *μ*M) reduced the level of p-P38 by 78.72%. Furthermore, INDO (10 *μ*M) reduced the level of p-ERK (by 58.19%), p-JNK (by 70.11%), and p-P38 (by 55.20%). These results indicate that BA can inhibit the phosphorylation of proteins involved in MAPK signaling in a dose-dependent manner.

### 3.8. Molecular Docking Analysis Reveals the Binding Mode between BA and TLR4-MD2 Complex

**Figure 7:**
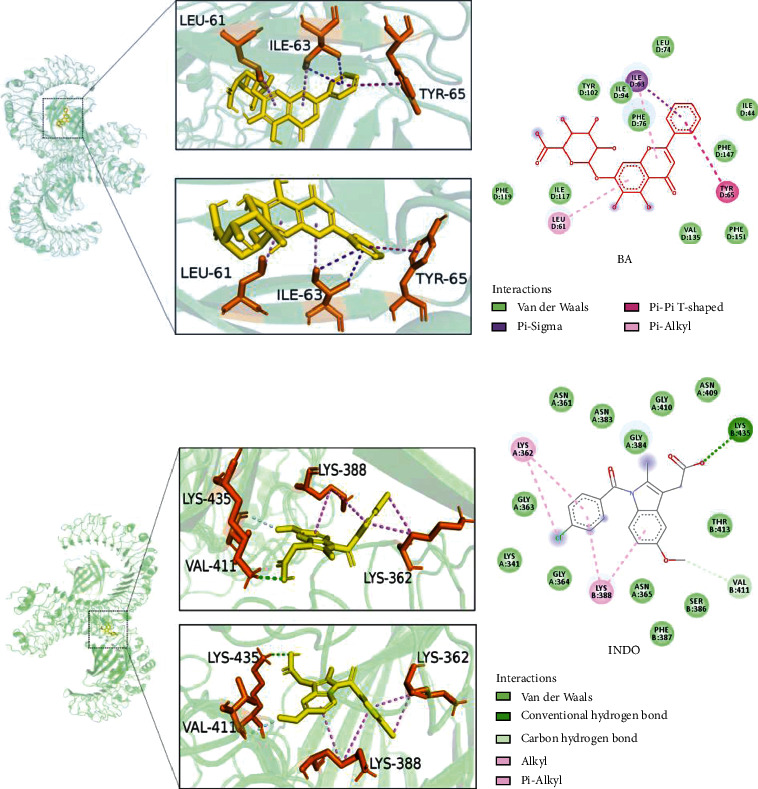
Molecular docking analysis of BA and INDO on TLR4-MD2 complex. The 3D and 2D views of the sites of BA in the TLR4-MD2 complex (a, b). The 3D and 2D views of the sites of INDO in the TLR4-MD2 complex (c, d).

BA showed a favorable conformation at the active site of TLR4-MD2 with the energy of −13.94 kJ/mol. The aromatic ring of BA is connected with amino acids Lie 63 of TLR4-MD2 by *π*-*σ* interaction (bonding distances of 3.93 Å) and connected with amino acids Tyr 65 of TLR4-MD2 by forming *π*-*π* T-shaped interaction (bonding distances of 5.33 Å). BA also could bind to Leu 61 of TLR4-MD2 by *π*-alkyl interaction, bonding distances of 5.24 Å (Figures 7(a) and 7(b)). INDO, as TLR4 antagonist, also adopted optimal conformation at the active site of TLR4-MD2 with the minimum energy of −27.42 kJ/mol. The carbonyl oxygen of INDO interacted with amino acids Lys 435 of TLR4-MD2 forming conventional hydrogen bond (H-bond), bonding distances of 2.29 Å. The aromatic ring of INDO is connected with amino acids Lys 362 and Lys 388 of TLR4-MD2 by *π*-alkyl interaction (bonding distances of 4.69 Å and 4.79 Å, respectively). INDO also could bind to Val 411 of TLR4-MD2 by carbon hydrogen bond (Figures 7(c) and 7(d)). These results indicated that BA could bind to the TLR4 receptor and participated in a powerful and stable interaction with the active site of TLR4-MD2.

## 4. Discussion

Microglial cells are the resident immuno competent cells in CNS, and inflammation mediated by microglia is closely associated with neurological disorders [18]. Anti-inflammatory treatment which targeted microglia could be a promising therapy for multiple neurodegenerative conditions [19]. BA, an anti-inflammatory flavonoid from the Radix of *S. baicalensis*, is excellent for the prevention of neurodegenerative diseases by its potent neuroprotective effects [20]. BA also makes a show of therapeutic effect on liver inflammation by blocking NF-*κ*B signal transduction [21]. In addition, BA can alleviate oxygen-glucose-deprived challenged microglia injury in ischemic diseases by attenuating expression of inflammatory cytokines TNF-*α*, IL-1*β*, IL-6, and IL-8 through TLR4 pathway [22]. Furthermore, A*β*-induced microglia activation is inhibited by BA through the JAK2/STAT3 signaling [23]. BA could also suppress the polarization and inflammatory injury of microglia by suppressing the 5-LOX/LTB4 pathway in BV-2 [24]. BA has showed potential therapeutic effect for depression by reducing the levels of proinflammatory factor IL-1*β* possibly through regulation of SIRT1-NF-*κ*B pathway in BV-2 microglia [25]. Further, BA improves neuroinflammation-induced depression-like behavior by inhibiting the expression of TLR4 through PI3K/Akt/FoxO1 signals [26]. Acute neurocognitive impairment and neuroinflammation in LPS-challenged mice are ameliorated by BA via a SIRT1/HMGB1-dependent pathway [27]. In the current study, we investigated the antineuroinflammatory effects of BA in microglia and further explored the effect of BA on TLR4/MyD88/NF-*κ*B and MAPK pathways. Present results indicate that BA can inhibit the production of proinflammatory mediators and cytokines, e.g., NO, PGE2, COX-2, and IL-1*β*, in LPS-induced microglia. These effects were attendant on the downregulation of miR-155 and several proteins in TLR4 signaling pathway.

The neuroprotective effect of BA is closely associated with the downregulation of the expression of iNOS and COX-2 [9, 11]. Therefore, we first assessed the effects of BA on the representative proinflammatory mediators and cytokines in LPS-stimulated BV-2 cells and found that BA can inhibit the release of NO and PGE2 by downregulating the levels of iNOS and COX-2. NO is a key regulatory molecule involved in a train of physiological and pathological processes, e.g., inflammatory and neurodegenerative diseases. INOS is responsible for the production of NO in different cells by converting L-arginine to L-citrulline [28, 29]. COX-2 can promote the release of arachidonic acid (AA) from membrane glycerol phospholipids and oxidized AA to proinflammatory eicosenoic acid (PGE2, leukotrienes, and thromboxanes), aggravating neuroinflammatory events in the brain. Overproduction of PGE2 can bring about the upregulation of cytokines, growth factors, and proinflammatory molecules [15, 30]. Similarly, IL-1*β* is another neuroimmune mediator that participates directly in neurodegeneration [31]. Recent research has been suggested that people carrying IL-1*β* (1473C/G) genotype are more vulnerable to neuroinflammation, and further develop to AD [32]. In addition, binding of IL-1*β* with TLR can lead to the overexpression of transcription factors and long-term chronic inflammation in the brain [33]. Our data showed that BA can inhibit IL-1*β* expression in LPS-stimulated microglia. Multiple studies have shown that PGE2, COX-2, and IL-1*β* expression levels are significantly increased in AD patients' brain [34, 35]. Our results showed that BA can significantly reduce the release of NO, IL-1*β*, and PGE2 by downregulating the iNOS and COX-2 at the kinase activity or protein levels in LPS-induced microglia. These results indicate that BA could be potentially used for the treatment of neurodegenerative diseases. miR-155 shows a critical role in the progression of neurodegenerative diseases. According to one study, several TLR4 ligands could increase miR-155 expression via either MyD88-dependent or MyD88-independent signaling pathways [16]. Our result showed that pretreatment with BA decreases the expression of miR-155 in LPS-stimulated microglia.

TLR4 signaling-mediated neuroinflammation leads to secondary brain damage in ischemic stroke. TLR4 is expressed on the surface of BV-2 microglia. LPS is a common ligand of TLR4, which can activate TLR4 in microglia via MyD88-dependent and independent patterns. In MyD88-dependent manner, recruitment of MyD88 to TLR complexes contributes to the activation of IRAKs, including IRAK1 and IRAK4 [36]. Our present study showed that BA inhibits the expression of TLR4, MyD88, IRAK4, and IRAK1 in a dose-dependent manner, indicating that BA can target TLR4-mediated MyD88-dependent pathway. Furthermore, the molecular docking analysis indicated that BA could stably bind TLR4-MD2 via *π*-bond. As a downstream factor of TLR4/MyD88/IRAK, NF-*κ*B also shows a pivotal role in inflammation meditated by microglia. Blockade of NF-*κ*B transcription in the microglial nucleus leads to the downregulation of NO, PGE2, and related proinflammatory cytokines [37–39]. In the cytoplasm, phosphorylation of I*κ*B*α* results in its further ubiquitination and degradation, and consequently, NF-*κ*B dimers are released and translocated to the nucleus to activate the expression of target genes [40]. Our result showed that BA can inhibit NF-*κ*B upregulation and I*κ*B*α* degradation and increase the phosphorylation of I*κ*B*α* in LPS-stimulated BV-2 cells, indicating that BA can also target NF-*κ*B signaling pathway to inhibit inflammatory response in microglia. MAPKs including ERK, JNK, and P38 subfamilies play crucial roles in inflammatory response in microglia [41]. MAPKs are also involved in the LPS-stimulated overproduction of COX-2 and iNOS [41, 42]. We showed that BA can inhibit LPS-induced phosphorylation of P38, ERK, and JNK, indicating that MAPK can also be targeted by BA.

Indomethacin (INDO) is a kind of nonsteroidal anti-inflammatory drugs (NSAIDs). Studies have confirmed that NSAIDs can effectively reduce incidence rate and risk of AD and delay the disease progression [43, 44]. In this study, we used INDO as a positive control. Interestingly, our study showed that BA may play a similar role as NSAIDs in LPS-stimulated BV-2. Our results showed that INDO significantly reduced the release of inflammatory mediators and cytokines and the expression of miR-155 and multiple signaling proteins. In addition, our results showed that both BA and INDO can interact with TLR4-MD2 complex in molecular docking analysis. Therefore, TLR4 pathway may be the target of both NSAIDs and BA in microglia. BA may be a valuable therapeutic candidate for the treatment of neuroinflammation and neurodegenerative diseases.

**Figure 8:**
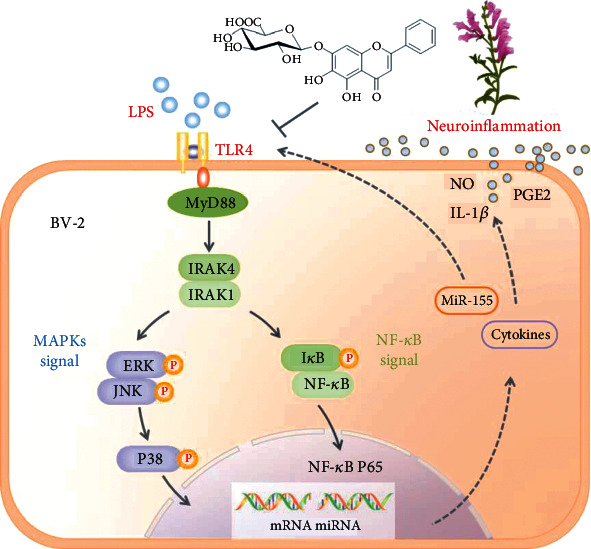
Mechanism of BA mitigating the neuroinflammation responses in LPS-stimulated BV-2 microglia. BA mitigates the neuroinflammation responses in LPS-stimulated BV-2 through the TLR4/MyD88/NF-*κ*B and MAPK pathway. BA inhibits the expression of miR-155 and release of inflammatory mediator (NO, PGE2, and IL-1*β*).

In current research, microglia generated abundant inflammatory meditators, NO, IL-1*β*, and PGE2 after being activated by LPS. The abnormal production and release of neuroimmune factors NO, PGE2, and IL-1*β* further aggravated the damage of neurons. While microglia are exposed to BA (1.5-22.5 *μ*M), the levels of inflammatory factors and the expression of related metabolic enzymes iNOS, COX-2, and miR-155 were reduced in a dose-dependent manner. As deeper research, the above changes were closely related with TLR4/MyD88/NF-*κ*B and MAPK pathway. After being combined with TLR4, BA suppressed the upregulation of TLR4, MyD88, IRAK4, IRAK1, P-I*κ*B*α*, and NF-*κ*B P65 and the downregulation of I*κ*B*α* in TLR4/MyD88/NF-*κ*B pathway and further inhibited the phosphorylation of ERK, JNK, and P38 of MAPK family (Figure 8). Finally, BA showed markedly antineuroinflammation effects in LPS-stimulated BV-2 microglia, which was similar to NSAID INDO.

In conclusion, this study demonstrated that BA exhibits anti-inflammatory function in LPS-stimulated BV-2 through inhibiting TLR4/NF-*κ*B and MAPK activation and may reduce the expression of neuroimmune mediator NO, PGE2, and IL-1*β*. BA may be a potential therapeutic drug for neuroinflammation-associated disorders in the future.

## Data Availability

The data presented in this study are available upon request from the corresponding author.

## Supplementary Materials

Supplementary Materials The supplementary materials include the original WB bands of iNOS/*β*-actin (Figure S1), COX-2/*β*-actin (Figure S2), TLR4/*β*-actin (Figure S3), MyD88/*β*-actin (Figure S4), IRAK4/*β*-actin (Figure S5), IRAK1/*β*-actin (Figure S6), NF-*κ*B P65/*β*-actin (Figure S7), p-I*κ*B*α*/*β*-actin (Figure S8), I*κ*B*α*/*β*-actin (Figure S9), p-P38/P38 (Figure S10), p-ERK/ERK (Figure S11), and p-JNK/JNK (Figure S12).

## References

[1] Hou Y. J., Dan X. L., Babbar M. (2019). Ageing as a risk factor for neurodegenerative disease. *Nature Reviews Neurology*.

[2] Stephenson J., Nutma E., van der Valk P., Amor S. (2018). Inflammation in CNS neurodegenerative diseases. *Immunology*.

[3] Deczkowska A., Keren-Shaul H., Weiner A., Colonna M., Schwartz M., Amit I. (2018). Disease-associated microglia: a universal immune sensor of neurodegeneration. *Cell*.

[4] Subhramanyam C. S., Wang C., Hu Q. D., Dheen S. T. (2019). Microglia-mediated neuroinflammation in neurodegenerative diseases. *Seminars in Cell & Developmental Biology*.

[5] Hansen D. V., Hanson J. E., Sheng M. (2018). Microglia in Alzheimer’s disease. *Journal of Cell Biology*.

[6] Ho M. S. (2019). Microglia in Parkinson’s disease. *Advances in Experimental Medicine and Biology*.

[7] Rodriguez-Gomez J. A., Kavanagh E., Engskog-Vlachos P. (2020). Microglia: agents of the CNS pro-inflammatory response. *Cell*.

[8] Rahimifard M., Maqbool F., Moeini-Nodeh S. (2017). Targeting the TLR4 signaling pathway by polyphenols: a novel therapeutic strategy for neuroinflammation. *Ageing Research Reviews*.

[9] Tu X. K., Yang W. Z., Shi S. S., Wang C. H., Chen C. M. (2009). Neuroprotective effect of baicalin in a rat model of permanent focal cerebral ischemia. *Neurochemical Research*.

[10] Heo H. J., Kim D. O., Choi S. J., Shin D. H., Lee C. Y. (2004). Potent inhibitory effect of flavonoids in *Scutellaria baicalensis* on amyloid *β* protein-induced neurotoxicity. *Journal of Agricultural and Food Chemistry*.

[11] Shih Y. T., Wu D. C., Liu C. M., Yang Y. C., Chen I. J., Lo Y. C. (2007). San-Huang-Xie-Xin-Tang inhibits _*Helicobacter pylori*_ -induced inflammation in human gastric epithelial AGS cells. *Journal of Ethnopharmacology*.

[12] Baskaran S. G., Sharp T. P., Sharp K. A. (2021). Computational graphics software for interactive docking and visualization of ligand-protein complementarity. *Journal of Chemical Information and Modeling*.

[13] Guterres H., Im W. (2020). Improving protein-ligand docking results with high-throughput molecular dynamics simulations. *Journal of Chemical Information and Modeling*.

[14] Wang G. S., Zhou B., Wang Z. Y. (2021). Pharmacological mechanisms underlying the anti-asthmatic effects of modified guomin decoction determined by network pharmacology and molecular docking. *Frontiers in Molecular Biosciences*.

[15] Patrignani P., Tacconelli S., Sciulli M. G., Capone M. L. (2005). New insights into COX-2 biology and inhibition. *Brain Research Reviews*.

[16] O'Connell R. M., Taganov K. D., Boldin M. P., Cheng G. H., Baltimore D. (2007). MicroRNA-155 is induced during the macrophage inflammatory response. *Proceedings of the National Academy of Sciences of the United States of America*.

[17] Wen Y., Zhang X. J., Dong L. P., Zhao J. R., Zhang C., Zhu C. H. (2015). Acetylbritannilactone modulates microRNA-155-mediated inflammatory response in ischemic cerebral tissues. *Molecular Medicine*.

[18] Rock R. B., Peterson P. K. (2006). Microglia as a pharmacological target in infectious and inflammatory diseases of the brain. *Journal of Neuroimmune Pharmacology*.

[19] Qian L., Flood P. M. (2008). Microglial cells and Parkinson’s disease. *Immunologic Research*.

[20] Sowndhararajan K., Deepa P., Kim M., Park S. J., Kim S. (2018). Neuroprotective and cognitive enhancement potentials of baicalin: a review. *Brain Sciences*.

[21] Cheng P., Wang T., Li W. (2017). Baicalin alleviates lipopolysaccharide-induced liver inflammation in chicken by suppressing TLR4-mediated NF-*κ*B pathway. *Frontiers in Pharmacology*.

[22] Hou J. C., Wang J., Zhang P. (2012). Baicalin attenuates proinflammatory cytokine production in oxygen-glucose deprived challenged rat microglial cells by inhibiting TLR4 signaling pathway. *International Immunopharmacology*.

[23] Xiong J. X., Wang C. Z., Chen H. Y. (2014). A*β*-induced microglial cell activation is inhibited by baicalin through the JAK2/STAT3 signaling pathway. *International Journal of Neuroscience*.

[24] Li H., Wang Y., Wang B. (2021). Baicalin and geniposide inhibit polarization and inflammatory injury of OGD/R-treated microglia by suppressing the 5-LOX/LTB4 pathway. *Neurochemical Research*.

[25] Yu H. Y., Zhang F. F., Guan X. D. (2019). Baicalin reverse depressive-like behaviors through regulation SIRT1-NF-kB signaling pathway in olfactory bulbectomized rats. *Phytotherapy Research*.

[26] Guo L. T., Wang S. Q., Su J. (2019). Baicalin ameliorates neuroinflammation-induced depressive-like behavior through inhibition of toll-like receptor 4 expression via the PI3K/AKT/FoxO1 pathway. *Journal of Neuroinflammation*.

[27] Li Y., Liu T. T., Li Y. T. (2020). Baicalin ameliorates cognitive impairment and protects microglia from LPS-induced neuroinflammation via the SIRT1/HMGB1 pathway. *Oxidative Medicine and Cellular Longevity*.

[28] Ghasemi M., Mayasi Y., Hannoun A., Eslami S. M., Carandang R. (2018). Nitric oxide and mitochondrial function in neurological diseases. *Neuroscience*.

[29] Tewari D., Sah A. N., Bawari S. (2021). Role of nitric oxide in neurodegeneration: function, regulation, and inhibition. *Current Neuropharmacology*.

[30] Kumar A., Behl T., Jamwal S., Kaur I., Sood A., Kumar P. (2020). Exploring the molecular approach of COX and LOX in Alzheimer’s and Parkinson’s disorder. *Molecular Biology Reports*.

[31] Yang K. C., Liu M. N., Liou Y. J., Hu L. Y., Yang B. H., Chou Y. H. (2021). Interleukin-1 family and serotonin transporter in first-episode, drug-naive major depressive disorder: a pilot study. *Journal of Psychiatric Research*.

[32] Babic Leko M., Nikolac Perkovic M., Klepac N. (2020). IL-1*β*, IL-6, IL-10, and TNF*α* single nucleotide polymorphisms in human influence the susceptibility to Alzheimer’s disease pathology. *Journal of Alzheimer’s Disease*.

[33] Sheng J. G., Jones R. A., Zhou X. Q. (2001). Interleukin-1 promotion of MAPK-p38 overexpression in experimental animals and in Alzheimer's disease: potential significance for tau protein phosphorylation. *Neurochemistry International*.

[34] Xiang Z. M., Ho L., Valdellon J. (2002). Cyclooxygenase (COX)-2 and cell cycle activity in a transgenic mouse model of Alzheimer's Disease neuropathology. *Neurobiology of Aging*.

[35] Tan Z. S., Beiser A. S., Vasan R. S. (2007). Inflammatory markers and the risk of alzheimer disease: the Framingham study. *Neurology*.

[36] Leitner G. R., Wenzel T. J., Marshall N., Gates E. J., Klegeris A. (2019). Targeting toll-like receptor 4 to modulate neuroinflammation in central nervous system disorders. *Expert Opinion on Therapeutic Targets*.

[37] Zhang J. W., Zheng Y. L., Luo Y., Du Y., Zhang X. J., Fu J. L. (2019). Curcumin inhibits LPS-induced neuroinflammation by promoting microglial M2 polarization via TREM2/ TLR4/ NF-*κ*B pathways in BV2 cells. *Molecular Immunology*.

[38] Ogunrinade F. A., Guetchueng S. T., Katola F. O. (2021). Zanthoxylum zanthoxyloidesinhibits lipopolysaccharide- and synthetic hemozoin-induced neuroinflammation in BV-2 microglia: roles of NF-*κ*B transcription factor and NLRP3 inflammasome activation. *Journal of Pharmacy and Pharmacology*.

[39] Lim H. S., Kim Y. J., Kim B. Y., Park G., Jeong S. J. (2018). The anti-neuroinflammatory activity of tectorigenin pretreatment via downregulated NF-*κ*B and ERK/JNK pathways in BV-2 microglial and microglia inactivation in mice with lipopolysaccharide. *Frontiers in Pharmacology*.

[40] Wang S., Wang H., Guo H., Kang L., Gao X., Hu L. (2011). Neuroprotection of scutellarin is mediated by inhibition of microglial inflammatory activation. *Neuroscience*.

[41] Chan C. K., Tan L. T. H., Andy S. N., Kamarudin M. N. A., Goh B. H., Kadir H. A. (2017). Anti-neuroinflammatory activity of *Elephantopus scaber* L. via activation of Nrf2/HO-1 signaling and inhibition of p38 MAPK pathway in LPS-induced microglia BV-2 cells. *Frontiers in Pharmacology*.

[42] Jin M. H., Chen D. Q., Jin Y. H., Han Y. H., Sun H. N., Kwon T. (2021). Hispidin inhibits LPS-induced nitric oxide production in BV-2 microglial cells via ROS-dependent MAPK signaling. *Experimental and Therapeutic Medicine*.

[43] Szekely C. A., Thorne J. E., Zandi P. P. (2004). Nonsteroidal anti-inflammatory drugs for the prevention of Alzheimer’s disease: a systematic review. *Neuroepidemiology*.

[44] Guan P. P., Yang L. Q., Xu G. B., Wang P. (2021). Indomethacin disrupts the formation of *β*-amyloid plaques via an *α*2-macroglobulin-activating lrp1-dependent mechanism. *International Journal of Molecular Sciences*.

