# Supplementary material for: Baicalin Mitigates the Neuroinflammation through the TLR4/MyD88/NF-κB and MAPK Pathways in LPS-Stimulated BV-2 Microglia
Source: Biomed Res Int. 2022 Nov 9;2022:3263446. doi: 10.1155/2022/3263446 (PMC9668451; doi:10.1155/2022/3263446)
Supplement: Supplementary Materials — The supplementary materials include the original WB bands of iNOS/β-actin (Figure S1), COX-2/β-actin (Figure S2), TLR4/β-actin (Figure S3), MyD88/β-actin (Figure S4), IRAK4/β-actin (Figure S5), IRAK1/β-actin (Figure S6), NF-κB P65/β-actin (Figure S7), p-IκBα/β-actin (Figure S8), IκBα/β-actin (Figure S9), p-P38/P38 (Figure S10), p-ERK/ERK (Figure S11), and p-JNK/JNK (Figure S12). [file 3263446.f1.pdf]

Supplementary Material

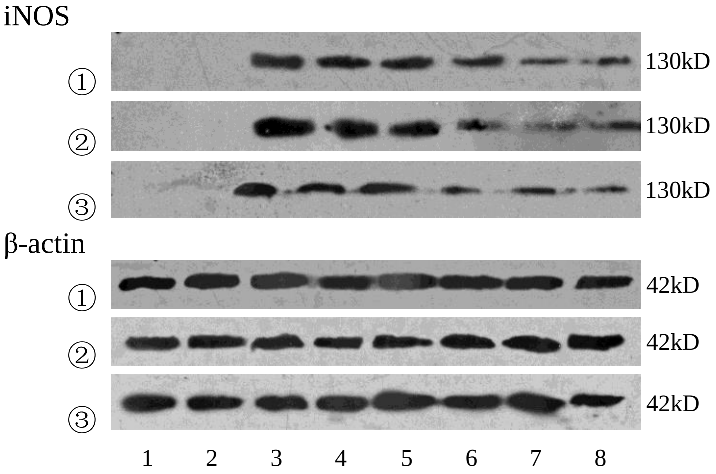

1: Control; 2: DMSO; 3: LPS (0.1 μg/mL); 4: BA (2.5 μM) + LPS (0.1 μg/mL); 5: BA (7.5 μM) + LPS (0.1 μg/mL); 6: BA (22.5 μM) + LPS (0.1 μg/mL); 7: NS-398 (10 μM) + LPS (0.1 μg/mL); 8: INDO (10 μM) + LPS (0.1 μg/mL)

Figure S1 Original WB bands of iNOS/β-actin

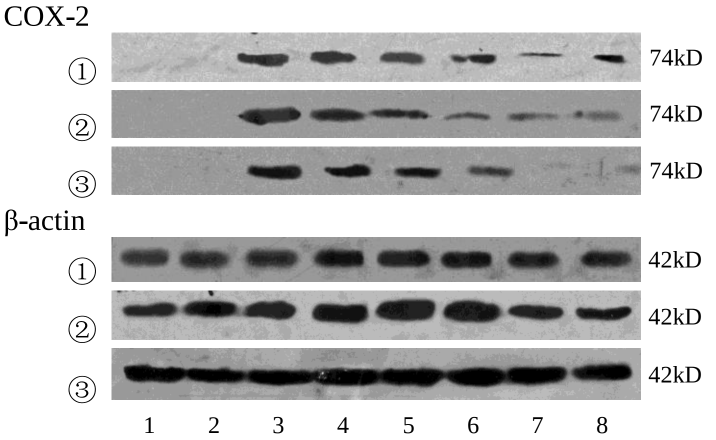

1: Control; 2: DMSO; 3: LPS (0.1 μg/mL); 4: BA (2.5 μM) + LPS (0.1 μg/mL); 5: BA (7.5 μM) + LPS (0.1 μg/mL); 6: BA (22.5 μM) + LPS (0.1 μg/mL); 7: NS-398 (10 μM) + LPS (0.1 μg/mL); 8: INDO (10 μM) + LPS (0.1 μg/mL)

Figure S2 Original WB bands of COX-2/β-actin

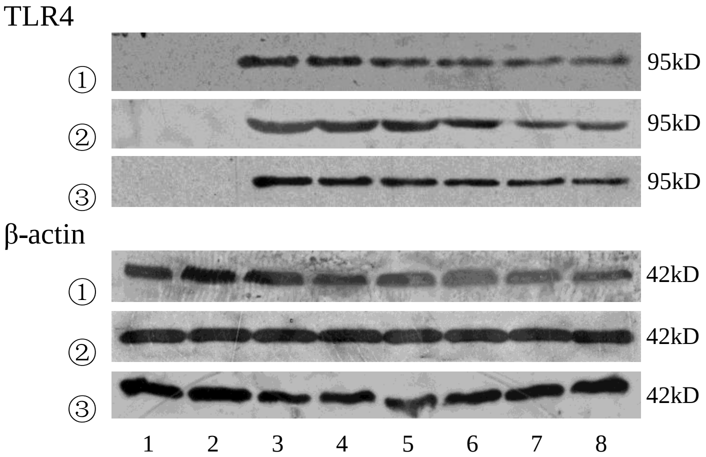

1: Control; 2: DMSO; 3: LPS (0.1 μg/mL); 4: BA (2.5 μM) + LPS (0.1 μg/mL); 5: BA (7.5 μM) + LPS (0.1 μg/mL); 6: BA (22.5 μM) + LPS (0.1 μg/mL); 7: MTS510 (1 μg/mL) + LPS (0.1 μg/mL); 8: INDO (10 μM) + LPS (0.1 μg/mL)

Figure S3 Original WB bands of TLR4/β-actin

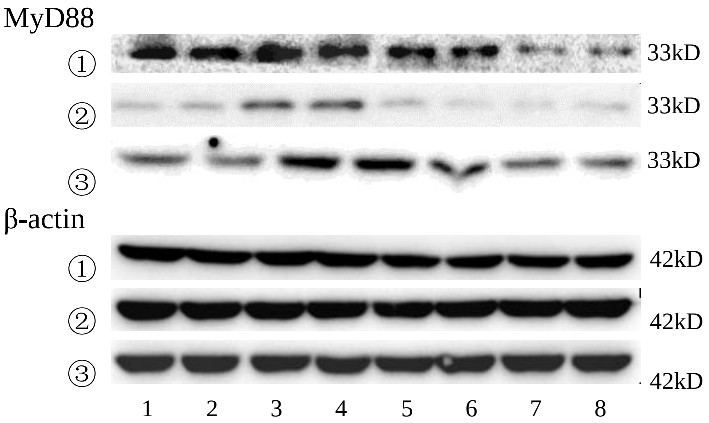

1: Control; 2: DMSO; 3: LPS (0.1 μg/mL); 4: BA (2.5 μM) + LPS (0.1 μg/mL); 5: BA (7.5 μM) + LPS (0.1 μg/mL); 6: BA (22.5 μM) + LPS (0.1 μg/mL); 7: ST2825 (0.1 μg/mL) + LPS (0.1 μg/mL); 8: INDO (10 μM)+ LPS (0.1 μg/mL)

Figure S4 Original WB bands of MyD88/β-actin

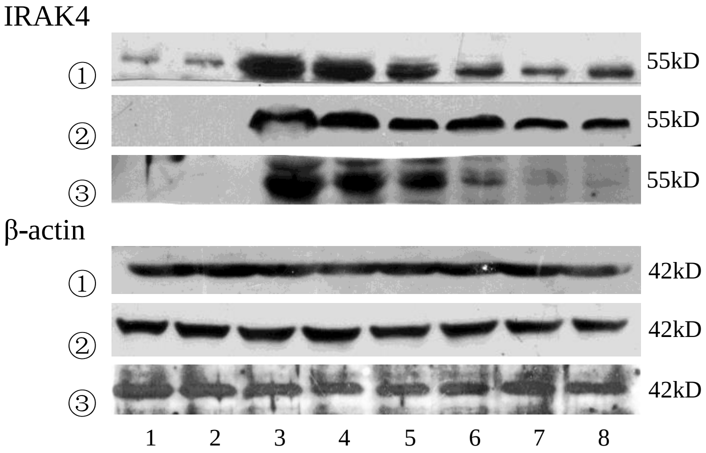

1: Control; 2: DMSO; 3: LPS (0.1 μg/mL); 4: BA (2.5 μM) + LPS (0.1 μg/mL); 5: BA (7.5 μM) + LPS (0.1 μg/mL); 6: BA (22.5 μM) + LPS (0.1 μg/mL); 7: IRAK-1/4 Inhibitor (0.1 μg/mL) + LPS (0.1 μg/mL); 8: INDO (10 μM) + LPS (0.1 μg/mL)

Figure S5 Original WB bands of IRAK4/β-actin

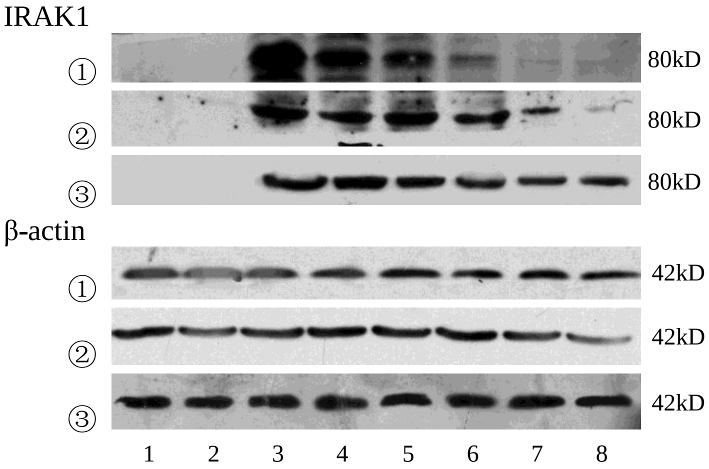

1: Control; 2: DMSO; 3: LPS (0.1 μg/mL); 4: BA (2.5 μM) + LPS (0.1 μg/mL); 5: BA (7.5 μM) + LPS (0.1 μg/mL); 6: BA (22.5 μM) + LPS (0.1 μg/mL); 7: IRAK-1/4 Inhibitor (0.1 μg/mL) + LPS (0.1 μg/mL); 8: INDO (10 μM)+ LPS (0.1 μg/mL)

Figure S6 Original WB bands of IRAK1/β-actin

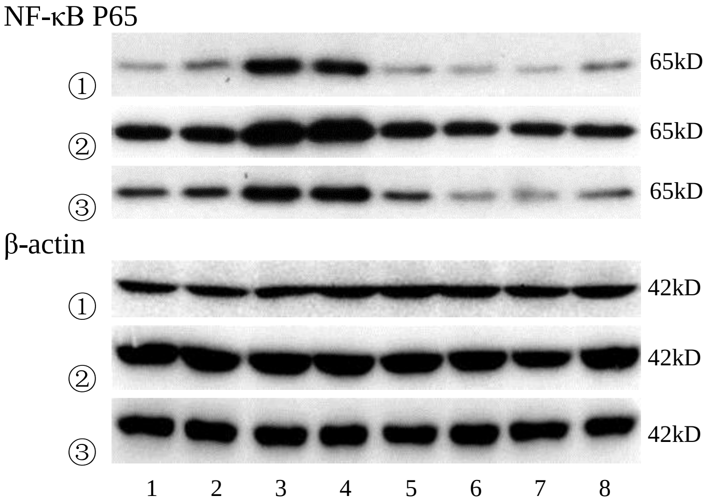

1: Control; 2: DMSO; 3: LPS (0.1 μg/mL); 4: BA (2.5 μM) + LPS (0.1 μg/mL); 5: BA (7.5 μM) + LPS (0.1 μg/mL); 6: BA (22.5 μM) + LPS (0.1 μg/mL); 7: PDTC (10 μM) + LPS (0.1 μg/mL); 8: INDO (10 μM)+ LPS (0.1 μg/mL)

Figure S7 Original WB bands of NF-κB P65/β-actin

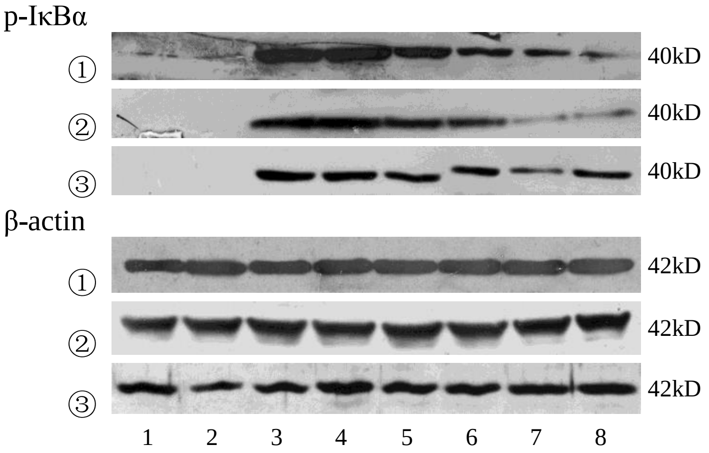

1: Control; 2: DMSO; 3: LPS (0.1 μg/mL); 4: BA (2.5 μM) + LPS (0.1 μg/mL); 5: BA (7.5 μM) + LPS (0.1 μg/mL); 6: BA (22.5 μM) + LPS (0.1 μg/mL); 7: PDTC (10 μM) + LPS (0.1 μg/mL); 8: INDO (10 μM) + LPS (0.1 μg/mL)

Figure S8 Original WB bands of p-IκBα/β-actin

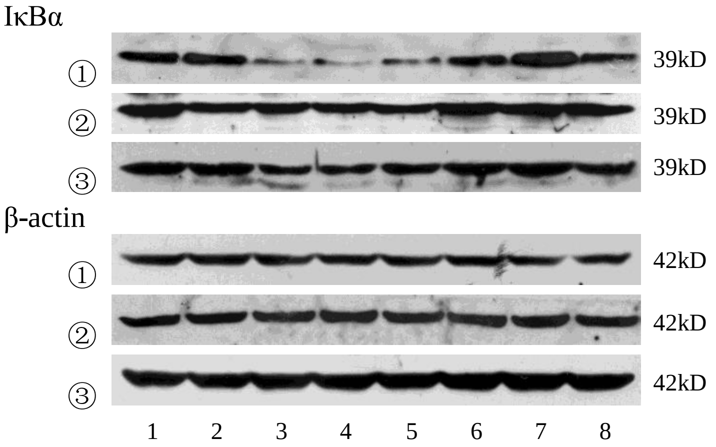

1: Control; 2: DMSO; 3: LPS (0.1 μg/mL); 4: BA (2.5 μM) + LPS (0.1 μg/mL); 5: BA (7.5 μM) + LPS (0.1 μg/mL); 6: BA (22.5 μM) + LPS (0.1 μg/mL); 7: PDTC (10 μM) + LPS (0.1 μg/mL); 8: INDO (10 μM) + LPS (0.1 μg/mL)

Figure S9 Original WB bands of IκBα/β-actin

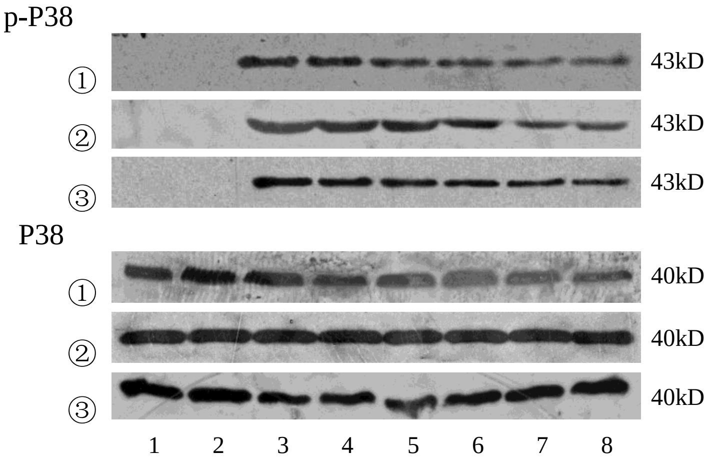

1: Control; 2: DMSO; 3: LPS (0.1 μg/mL); 4: BA (2.5 μM) + LPS (0.1 μg/mL); 5: BA (7.5 μM) + LPS (0.1 μg/mL); 6: BA (22.5 μM) + LPS (0.1 μg/mL); 7: SB203580 (25 μM) + LPS (0.1 μg/mL); 8: INDO (10 μM) + LPS (0.1 μg/mL)

Figure S10 Original WB bands of p-P38/P38

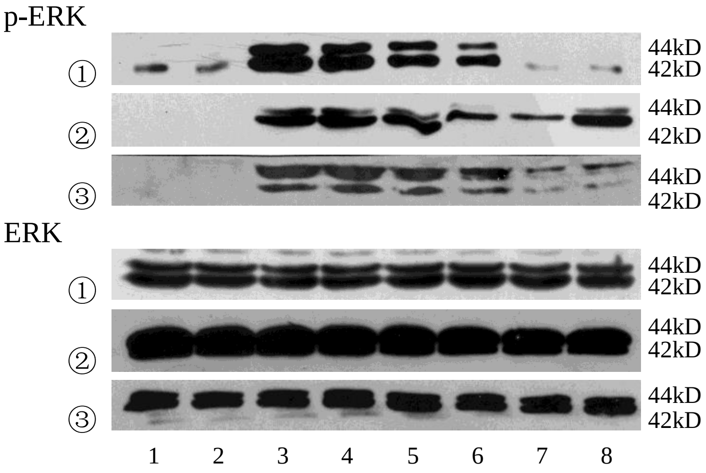

1: Control; 2: DMSO; 3: LPS (0.1 μg/mL); 4: BA (2.5 μM) + LPS (0.1 μg/mL); 5: BA (7.5 μM) + LPS (0.1 μg/mL); 6: BA (22.5 μM) + LPS (0.1 μg/mL); 7: U0126 (10 μM) + LPS (0.1 μg/mL); 8: INDO (10 μM) + LPS (0.1 μg/mL)

Figure S11 Original WB bands of p-ERK/ERK

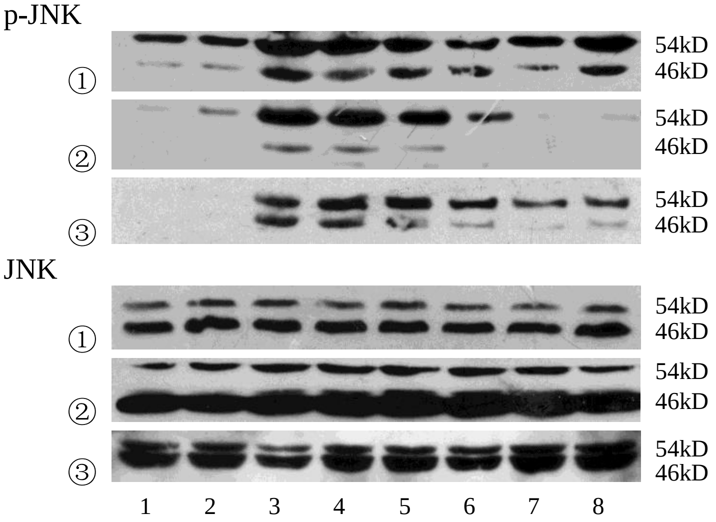

1: Control; 2: DMSO; 3: LPS (0.1 μg/mL); 4: BA (2.5 μM) + LPS (0.1 μg/mL); 5: BA (7.5 μM) + LPS (0.1 μg/mL); 6: BA (22.5 μM) + LPS (0.1 μg/mL); 7: SP600125 (25 μM) + LPS (0.1 μg/mL); 8: INDO (10 μM) + LPS (0.1 μg/mL)

Figure S12 Original WB bands of p-JNK/JNK
